# Neurotoxicity of immune checkpoint inhibitors: a retrospective pharmacovigilance study using FAERS database

**DOI:** 10.3389/fimmu.2026.1769013

**Published:** 2026-03-26

**Authors:** Lin Cao, Yanfei Wang, Peng Dai, Xia Li

**Affiliations:** 1 College of Traditional Chinese Medicine, Changchun University of Chinese Medicine, Jilin, Changchun, China; 2 Key Laboratory of Carcinogenesis and Translational Research (Ministry of Education), Day Oncology Unit, Peking University Cancer Hospital and Institute, Beijing, China

**Keywords:** combination therapy, CTLA-4, FAERS, immune checkpoint inhibitors (ICIs), neurological adverse events, PD-1/PD-L1

## Abstract

**Background:**

Immune checkpoint inhibitors (ICIs), an antitumor therapeutic strategy, have shown great potential for cancer treatment. With the widespread use of ICIs, immune-related adverse events (irAEs) have increased gradually. Neurotoxicity, as an irAE, has a low incidence rate, but high disability and mortality rates, warranting clinical attention.

**Material and methods:**

This study aimed to quantify the association between neurological adverse events and ICI treatment and describe the characteristics of ICI-related neurological complications in real-world practice. Data were sourced from the FDA Adverse Event Reporting System (FAERS) database from the first quarter of 2004 to the first quarter of 2025. Data mining was performed using the reporting odds ratio (ROR) method. Classification and statistics were conducted using the system organ class (SOC), high-level group term (HLGT), and preferred term (PT) from the Medical Dictionary for Regulatory Activities (MedDRA) terminology set (version 28.0).

**Results:**

The entire FAERS database included 56,321,150 adverse event records, of which 26,629 records were identified as being related to neurological immune-related adverse events (n-irAEs) following ICI therapy. The main adverse events include encephalitis, encephalopathy, myasthenia gravis, paraneoplastic syndromes, and peripheral neuropathy. Among these reports, males (53.51%) outnumbered females (37.82%), and the median patient age was 67 years. The number of reports in 2024 was slightly higher than that in other years, but the difference was not significant. Physicians were the primary reporters (43.23%) and the United States had the highest number of reports (35.78%). The median time to the onset of adverse events was 30 days. Serious reports accounted for a high proportion of the patients (91.23%).

**Conclusion:**

Pharmacovigilance analysis indicated that neurological immune-related adverse events associated with ICIs mainly involved central nervous system inflammation and neuromuscular junction dysfunction. Different ICI immunotherapies are associated with distinct neurological disease characteristics, and combination therapies may influence the reporting patterns of neurotoxicity. In clinical practice, the early recognition and management of ICI-related n-irAEs are critical.

## 1 Introduction

Immune checkpoint inhibitors (ICIs) bind to immune checkpoint proteins to relieve the tumor-induced suppression of T-cell function, thereby exerting antitumor effects (1). Current checkpoint inhibitors mainly include PD-1 inhibitors (pembrolizumab, nivolumab, toripalimab, tislelizumab, cemiplimab, and dostarlimab), PD-L1 inhibitors (atezolizumab, avelumab, durvalumab), and CTLA-4 antibodies (ipilimumab). The main adverse events associated with ICIs are concentrated in the skin, gastrointestinal tract, endocrine system, lungs, and musculoskeletal system, whereas neurological immune-related adverse events (n-irAEs) are relatively rare (2). Both the central and peripheral nervous systems can be affected (3). However, adverse neurological events can sometimes be severe and even fatal (4), and their pathogenesis remains unclear. Peripheral nervous system diseases account for 75-83% of n-irAEs, while central nervous system (CNS) diseases account for 17-25% (5). The diseases primarily include, peripheral nervous system disorders: peripheral neuropathy, neuromuscular junction dysfunction, myopathy/myositis and CNS disorders: encephalopathy/encephalitis, demyelinating diseases (multiple sclerosis), meningitis, myelitis (6, 7). Given the extensive use of ICIs in clinical practice, to further understand the characteristics of ICI-related neurological adverse events and expand real-world data on new adverse events, we conducted a disproportionality analysis of ICI-related n-irAEs using the FAERS database to characterize and evaluate ICI regimen-related neurological toxicity.

## 2 Material and methods

### 2.1 Data source and processing

For this retrospective pharmacovigilance study, raw data were obtained from the FDA Adverse Event Reporting System (FAERS) database and downloaded from the US FDA website. The original ASCII data packages were downloaded for data mining, and SAS 9.4 was used for statistical analysis. As the database relies on spontaneous reporting, it contains duplicate reports or reports that have been withdrawn or deleted. Therefore, this study strictly followed the FDA guidance documents for data cleaning. The data cleaning rules were as follows: first, duplicate reports were removed according to the FDA-recommended method: select the PRIMARYID, CASEID, and FDA_DT fields from the DEMO table, sorted by CASEID, FDA_DT, and PRIMARYID; for reports with the same CASEID, keep the one with the largest FDA_DT value; for reports with identical CASEID and FDA_DT, keep the one with the largest PRIMARYID. Second, since the first quarter of 2019, each quarterly data package has included a list of deleted reports. After deduplication, the reports listed by CASEID in the deletion list were removed.

The WHO Drug Dictionary (March 2025 version) was used to standardize all drug names in the database (including trade names, generic names, ingredient names, and product codes). The standardized drug names were searched using the terms: “Pembrolizumab, Atezolizumab, Nivolumab, Ipilimumab, Durvalumab, Avelumab, Cemiplimab, Tislelizumab, Dostarlimab, Toripalimab.” Adverse event names in the FAERS database were coded according to the Medical Dictionary for Regulatory Activities (MedDRA) dictionary (version 28.0). The MedDRA dictionary was used to recode the Preferred Term names in the FAERS database and to obtain system organ class (SOC), high-level group term (HLGT), and preferred term (PT) for subsequent analysis. All PTs that are classified under the ‘Nervous system disorders’ SOC were included as n-irAEs).

### 2.2 Statistical analysis

This study employed the reporting odds ratio (ROR) method to detect adverse drug event signals associated with ICIs. ROR can identify consistent adverse drug event (ADE) patterns across different studies, providing valuable evidence for drug safety assessments and clinical decision-making. The criteria for signal generation were: the number of reported target ADE events (a) ≥ 3 and the lower limit of the 95% confidence interval > 1. The ROR value reflects the magnitude of the statistical association between ICIs and ADEs in the reporting database; a higher ROR indicates a stronger disproportionality signal, rather than a higher clinical risk. The ROR calculation formula is as follows:


$$ROR=\frac{\left(a/c\right)}{\left(b/d\right)}\;=\frac{ad}{bc}$$

$$SE\left(InROR\right)=\sqrt{\left(\frac{1}{a}+\frac{1}{b}+\frac{1}{c}+\frac{1}{d}\right)}$$

$$95\%CI={\text{e}}^{In\left(ROR\right)\pm 1.96\sqrt{\left(\frac{1}{a}+\frac{1}{b}+\frac{1}{c}+\frac{1}{d}\right)}}$$

Note: a: Number of reported adverse neurological disease events associated with ICIs.

b: Total number of adverse events other than neurological diseases associated with ICIs.

c: Number of cases of various neurological disease adverse events associated with drugs other than ICIs.

d: Number of adverse events other than various neurological diseases associated with drugs other than ICIs.

## 3 Results

### 3.1 Basic information on reports of neurotoxic adverse events induced by ICIs

The entire FAERS database included 56,321,150 adverse event records, of which 26,629 records were identified as being related to n-irAEs following ICI therapy. The main adverse events include encephalitis, encephalopathy, myasthenia gravis, paraneoplastic syndromes, and peripheral neuropathy.

Among these reports, males (53.51%) outnumbered females (37.82%), with the other 8.67% labeled as not specific. Adverse events occurred predominantly in the elderly population with a median age of 67 years.

**Figure 1:**
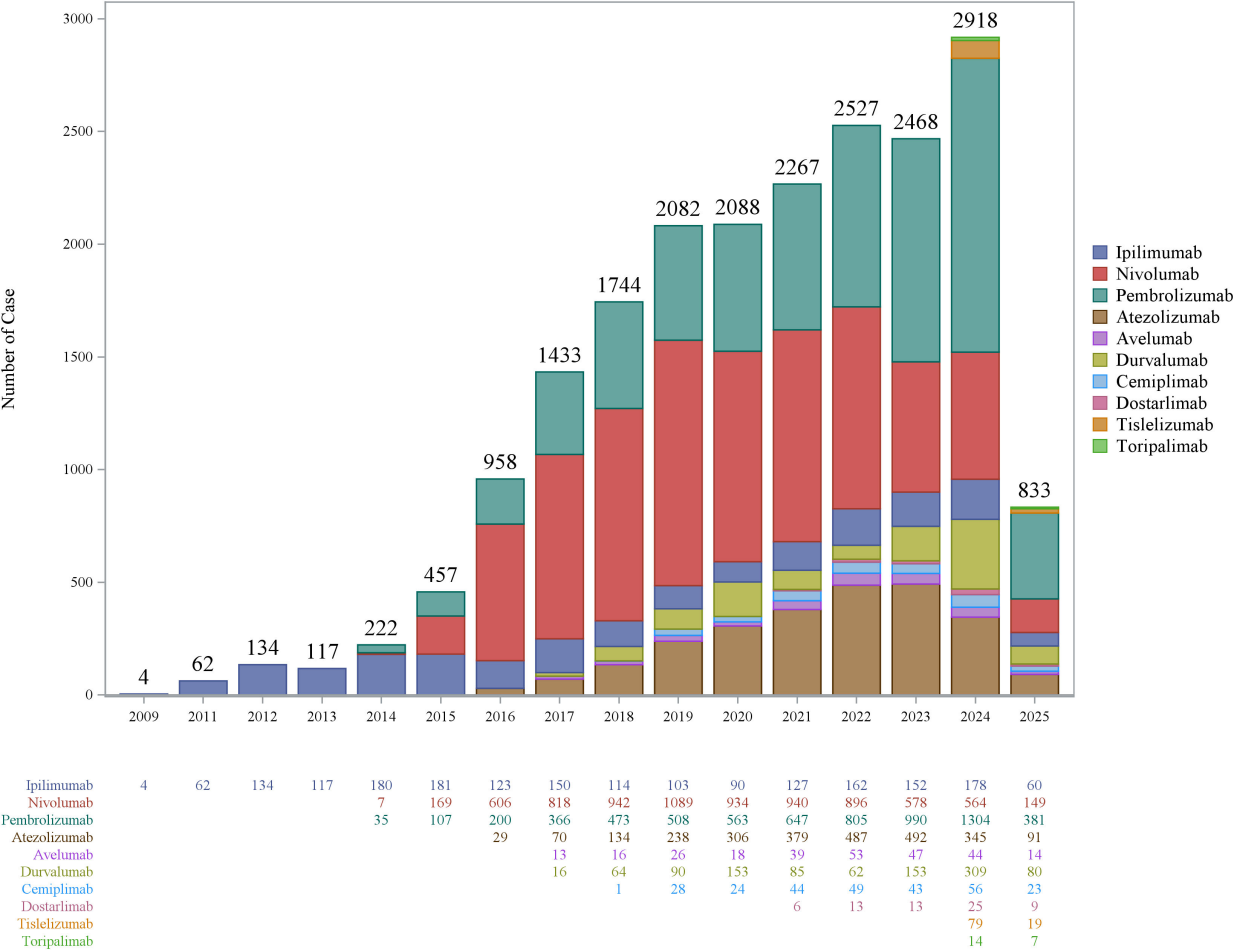
Annual distribution of immune checkpoint inhibitor (ICI)-induced neurotoxic adverse event (AE) reports. This figure shows the annual variation trend in the number of reports of neurotoxic adverse events associated with 10 immune checkpoint inhibitors (ICIs, including PD-1/PD-L1 and CTLA-4 inhibitors) from 2009 to 2025.

**Figure 2:**
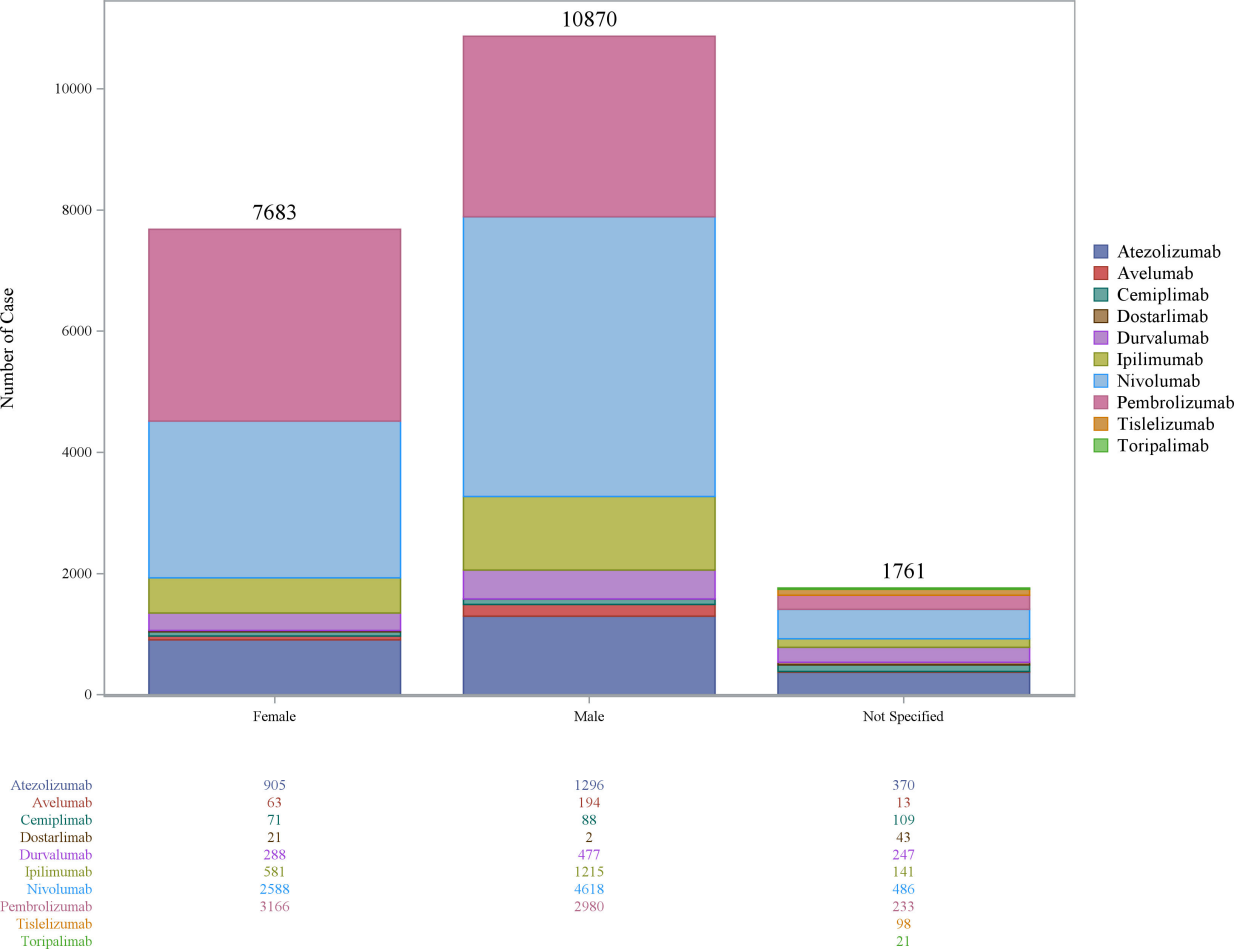
Sex distribution of neurological immune-related adverse event reports. This figure presents the sex distribution characteristics of reports of neurotoxic adverse events associated with 10 immune checkpoint inhibitors (ICIs).

Demographic data analysis showed that since 2009, reports of neurotoxic ADEs associated with ICIs have shown a continuous upward trend, particularly in 2024, reaching 2918 cases (14.36%), with pembrolizumab showing a particularly noticeable increasing trend (**Figure 1**). The number of ADE reports was slightly higher in males than in females, especially for Pembrolizumab, Nivolumab, and Ipilimumab (**Figure 2**). Health professionals were the primary reporters of this study. Reports have mainly originated in the United States and Japan. Indications were primarily lung cancer (3598, 17.67%), melanoma (3564, 17.51%), and renal cancer (1746, 8.59%). Serious adverse events accounted for 91.23%, with deaths comprising 17%, and life-threatening events accounting for 9.61%, indicating that ICI-related neurotoxic diseases have potentially life-threatening characteristics and warrant sufficient attention (**Table 1**).

**Table 1:** Patient characteristics of neurotoxic adverse events induced by immune checkpoint inhibitors.

Characteristics	Total no. (%)
Gender	
Female	7683 (37.82)
Male	10870 (53.51)
Not Specified	1761 (8.67)
Age (years)	
N (Missing)	15567 (4747)
Mean (SD)	65.10 (12.76)
Median (Q1, Q3)	67.00 (58.00, 74.00)
Min, Max	0.00,100.00
Reporting year	
2009	4 (0.02)
2011	62 (0.31)
2012	134 (0.66)
2013	117 (0.58)
2014	222 (1.09)
2015	457 (2.25)
2016	958 (4.72)
2017	1433 (7.05)
2018	1744 (8.59)
2019	2082 (10.25)
2020	2088 (10.28)
2021	2267 (11.16)
2022	2527 (12.44)
2023	2468 (12.15)
2024	2918 (14.36)
2025	833 (4.10)
Reporter	
Consumer	5590 (27.52)
Lawyer	10 (0.05)
Not Specified	224 (1.10)
Other health-professional	2027 (9.98)
Pharmacist	3681 (18.12)
Physician	8782 (43.23)
Reported countries (Top 10)	
United States of America	7269 (35.78)
Japan	4497 (22.14)
France	1347 (6.63)
Germany	998 (4.91)
China	769 (3.79)
Canada	678 (3.34)
United Kingdom	513 (2.53)
Italy	465 (2.29)
Australia	336 (1.65)
Spain	
Patients with serious and nonserious reports	306 (1.51)
Serious	18533 (91.23)
Non-Serious	1781 (8.77)
Outcome	
Life-Threatening	1952 (9.61)
Hospitalization - Initial or Prolonged	10352 (50.96)
Disability	1125 (5.54)
Death	3454 (17.00)
Congenital Anomaly	7 (0.03)
Required Intervention to Prevent Permanent	
Impairment/Damage	55 (0.27)
Other	15109 (74.38)
Indication	
Lung cancer	3598 (17.67)
Malignant melanoma	3564 (17.51)
Renal cell carcinoma	1746 (8.59)
Breast cancer	744 (3.63)
Hepatocellular carcinoma	690 (3.40)
Gastric cancer	413 (2.03)
Endometrial cancer	379 (1.86)
Bladder cancer	222 (1.07)
Esophageal carcinoma	212 (1.04)
Head and neck cancer	182 (0.89)
Time to onset (day)	
N (Missing)	8493 (11821)
Mean (SD)	87.35 (179.96)
Median (Q1, Q3)	30.00 (9.00,91.0)
Min, Max	0.00, 4070.00

SD, standard derivation; Q1, first quartile; Q3, third quartile.

Results were displayed as number (%) if not particularly stated.

### 3.2 PT signal detection results

**Figure 3:**
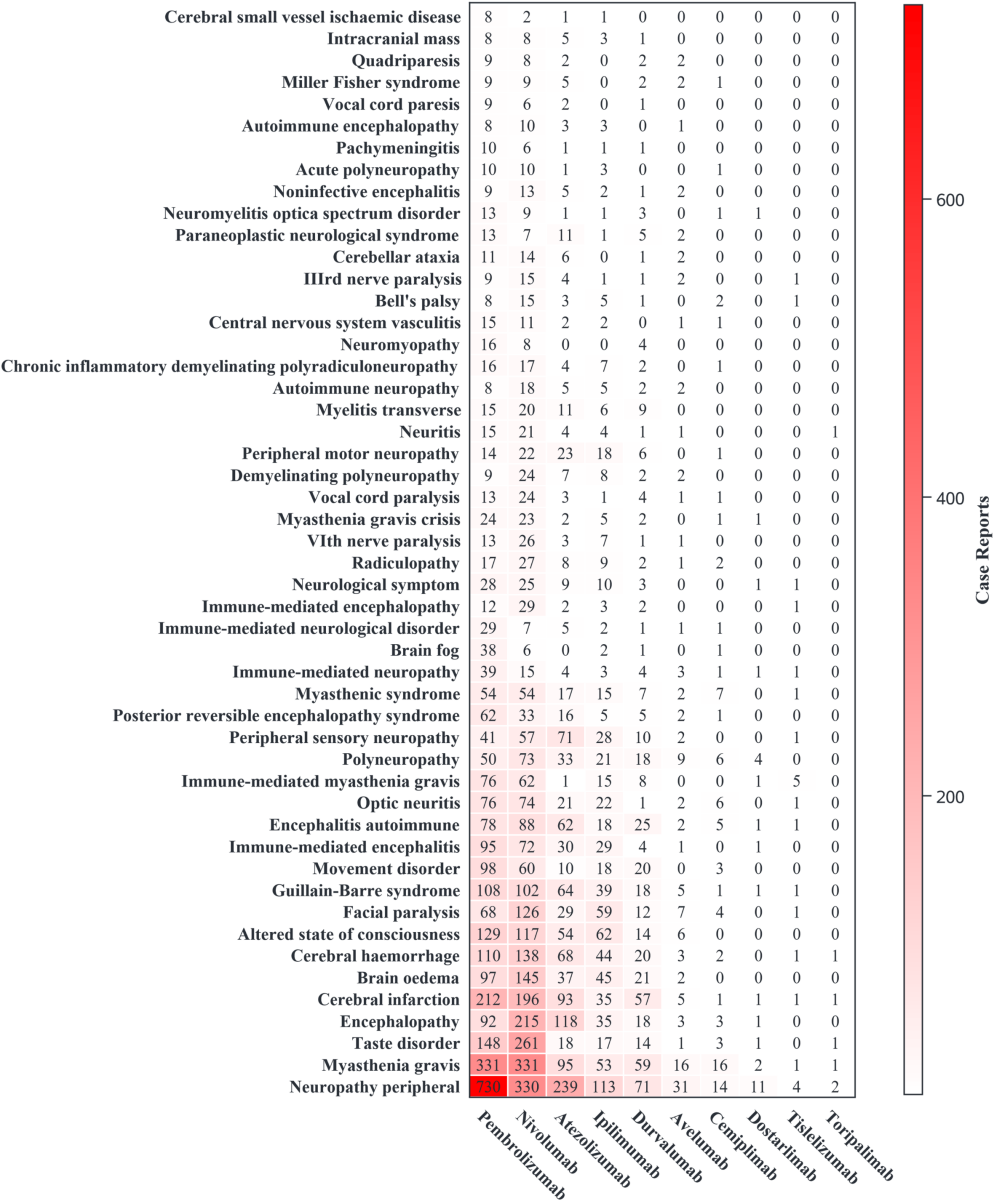
The number of reports for 50 specific Preferred Terms of neurological immune-related adverse events.

**Figure 4:**
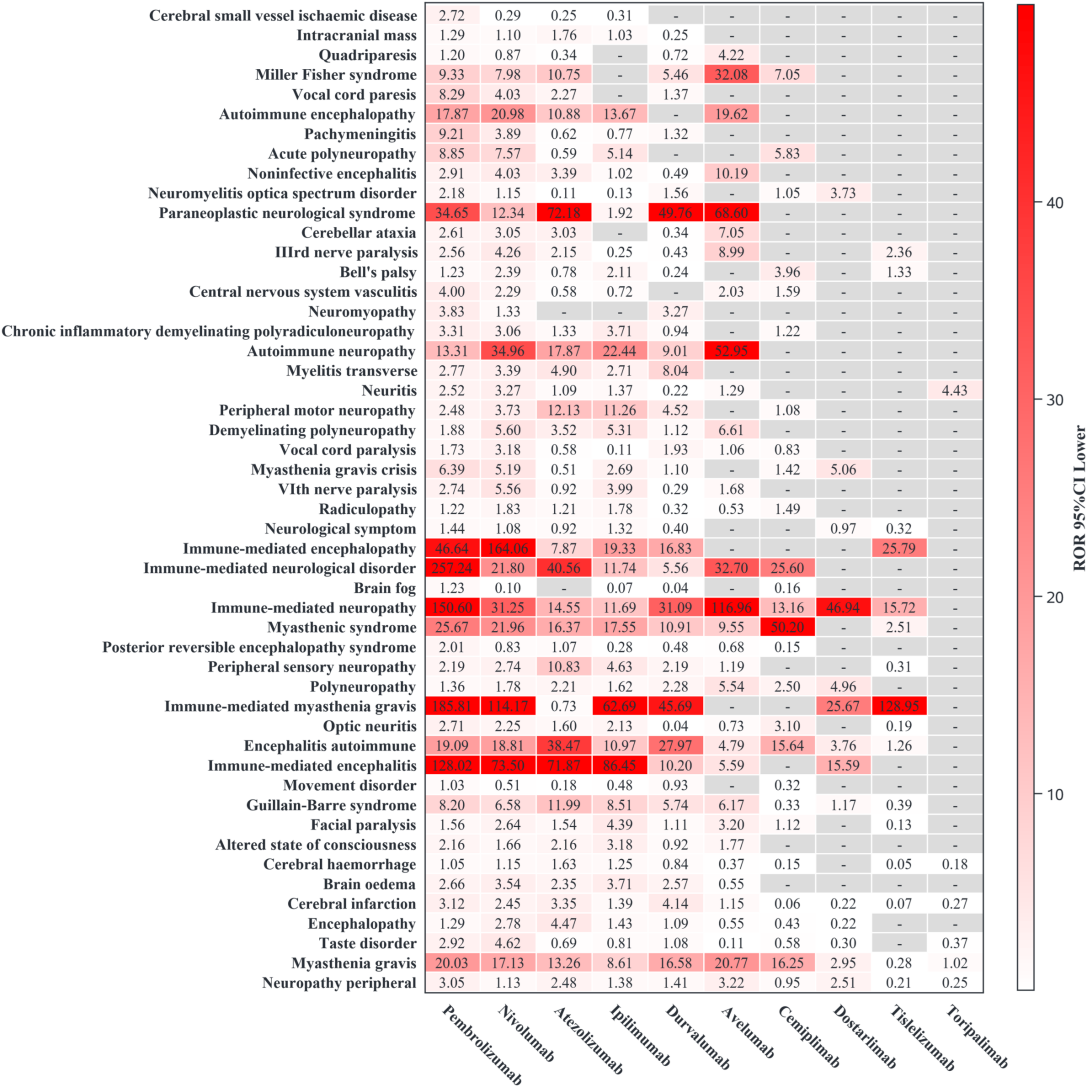
The signal intensity of 50 specific preferred terms of neurological immune-related adverse event reports. This figure analyzes the signal intensity of 50 specific Preferred Terms (PTs) for neurotoxicity using the Reporting Odds Ratio (ROR) method.

A total of 571 PTs within the ‘Nervous system disorders’ SOC were associated with ICIs. Among these, we identified 89 PTs with significant ROR signals (reports number≥3 and lower limit of 95% CI >1). We further selected 50 PTs with credible ROR signals, prioritizing those with the largest lower limits of the 95% confidence intervals of ROR (ROR_025_). The specific PT case counts and ROR signal strength results are shown in Figures (**Figures 3**, **4**).

Among the 50 selected PTs, the drugs with the highest number of ADE reports were pembrolizumab (3110, 38.61%), nivolumab (2989, 37.11%), and atezolizumab (1218, 15.12%). PTs with the highest reported counts were peripheral neuropathy (1545, 19.18%), myasthenia gravis (905, 11.24%), and cerebral infarction (602, 7.47%). CNS-related PTs mainly showed high signal strength for immune-mediated encephalitis and immune-mediated encephalopathy; PNS-related PTs showed high signal strength for immune-mediated myasthenia gravis and paraneoplastic neurological syndromes. Different ICIs were associated with different PT signal strengths. This study performed subgroup stratification and sensitivity analysis for drugs with higher case counts, including pembrolizumab, nivolumab, cemiplimab, atezolizumab, durvalumab, avelumab, and ipilimumab. Other drugs were not analyzed because of the small number of cases.

Among PD-1 class drugs, pembrolizumab showed high signals for immune-mediated neurological disorders and myasthenia gravis. Stratified by sex and age, the PT with the highest signal strength was an immune-mediated neurological disorder. In the sensitivity analysis excluding combination drugs, the PTs with the strongest association remained immune-mediated neurological disorders and immune-mediated myasthenia gravis. Nivolumab showed high signals for immune-mediated encephalopathy and immune-mediated myasthenia gravis. In sex stratification, the PTs with higher signals were immune-mediated myasthenia gravis and immune-mediated encephalopathy. In age stratification, the PTs with higher signals in young and middle-aged populations were immune-mediated encephalitis and immune-mediated encephalopathy, whereas in the elderly population, the PT with higher signal strength was immune-mediated myasthenia gravis. In sensitivity analyses excluding combination drugs, immune-mediated optic neuritis also emerged as a signal of interest. Cemiplimab showed high signal strength for Lambert-Eaton myasthenic syndrome and myasthenia gravis. According to sex and age stratification, the PT with high signal strength was myasthenia gravis. In the sensitivity analysis, the PT with the strongest association was Lambert-Eaton myasthenic syndrome. Tislelizumab showed a significant signal for immune-mediated myasthenia gravis. Dostarlimab showed signals for polyneuropathy and peripheral neuropathy. Toripalimab did not show PT-related signals, likely due to the limited number of cases.

Among PD-L1 class drugs, atezolizumab showed significant signals for paraneoplastic neurological syndromes and immune-mediated encephalitis. In sex stratification, the higher signal PT for males was paraneoplastic neurological syndrome, and for females, it was immune-mediated encephalitis, but the PT with a higher report count was peripheral neuropathy. In age stratification, the PT with a higher signal in young and middle-aged populations was immune-mediated encephalitis, whereas in the elderly population, the PT with a higher signal was paraneoplastic neurological syndrome. In the sensitivity analysis excluding combination drugs, the signals for autoimmune encephalitis and immune-mediated encephalitis remained significant. Durvalumab showed significant signals for paraneoplastic neurological syndromes and immune-mediated myasthenia gravis. In sex stratification, the higher signal PT for females was autoimmune encephalitis, and for males, it was a paraneoplastic neurological syndrome. In age stratification, the PT with a higher signal in middle-aged populations was autoimmune encephalitis, whereas in the elderly population, the PT with a higher signal was immune-mediated neuropathy. In the sensitivity analysis excluding combination drugs, the PT with the highest signal was myasthenia gravis. Avelumab showed a significant signal for myasthenia gravis. In sex stratification, the higher signal PT for females was paraneoplastic encephalomyelitis, and for males, it was myasthenia gravis. However, in the sensitivity analysis, the PT with a higher signal was associated with paraneoplastic encephalomyelitis.

Among the CTLA-4 drugs, ipilimumab showed significant signals for immune-mediated encephalitis and immune-mediated myasthenia gravis. In sex stratification, the PT with a higher signal was immune-mediated encephalitis. In the age stratification, the PT with a higher signal in middle age was immune-mediated optic neuritis, and in the elderly population, it was meningoradiculitis. In the sensitivity analysis, excluding other drugs, the PT with the highest signal was meningoradiculitis.

### 3.3 HLGT signal detection results

**Figure 5:**
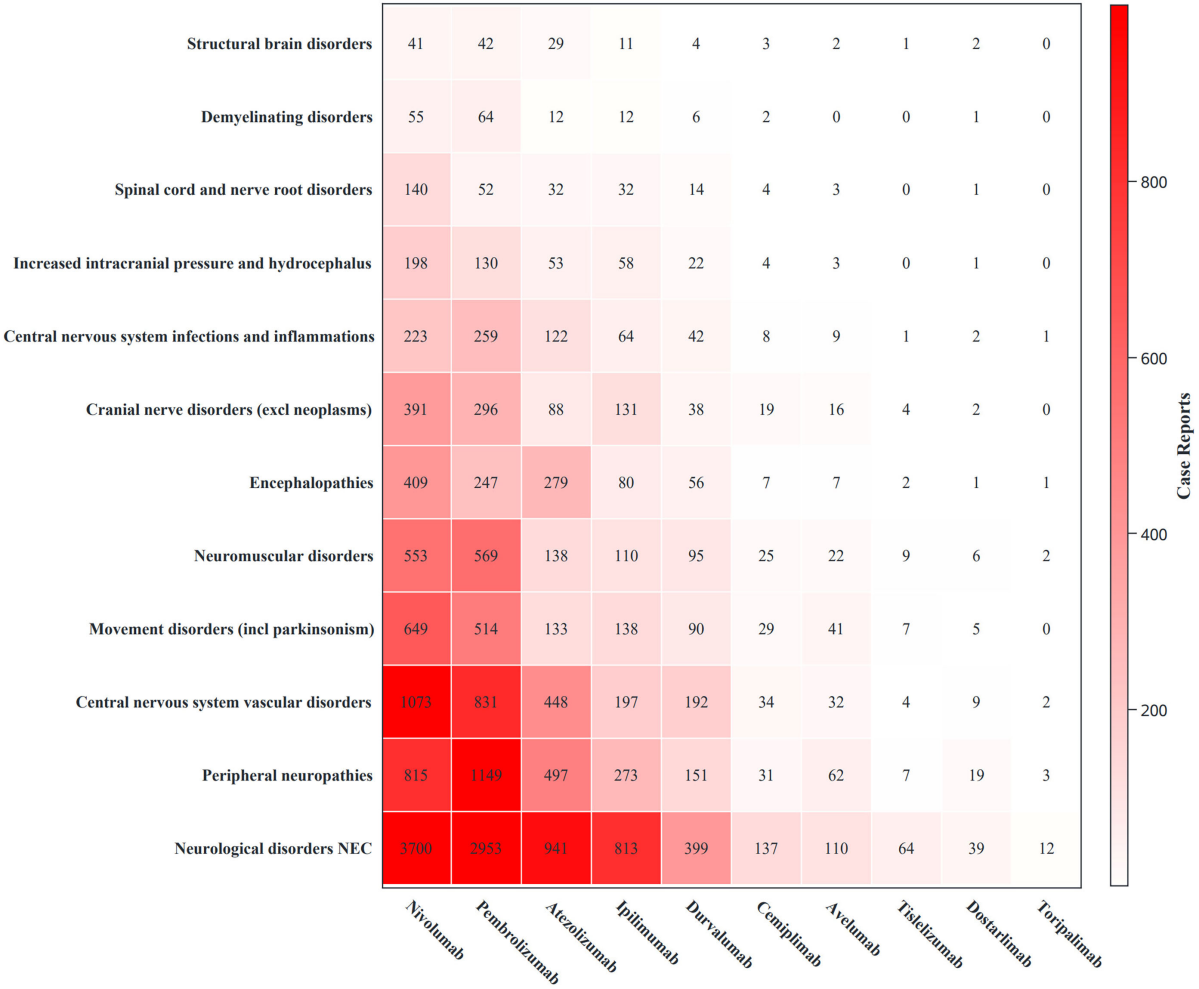
The number of reports for 12 specific high-level group terms of neurological immune-related adverse event reports. This figure quantifies the report numbers and compositional distribution of 12 specific High-Level Group Terms (HLGTs) related to immune checkpoint inhibitor (ICI)-induced neurotoxicity.

**Figure 6:**
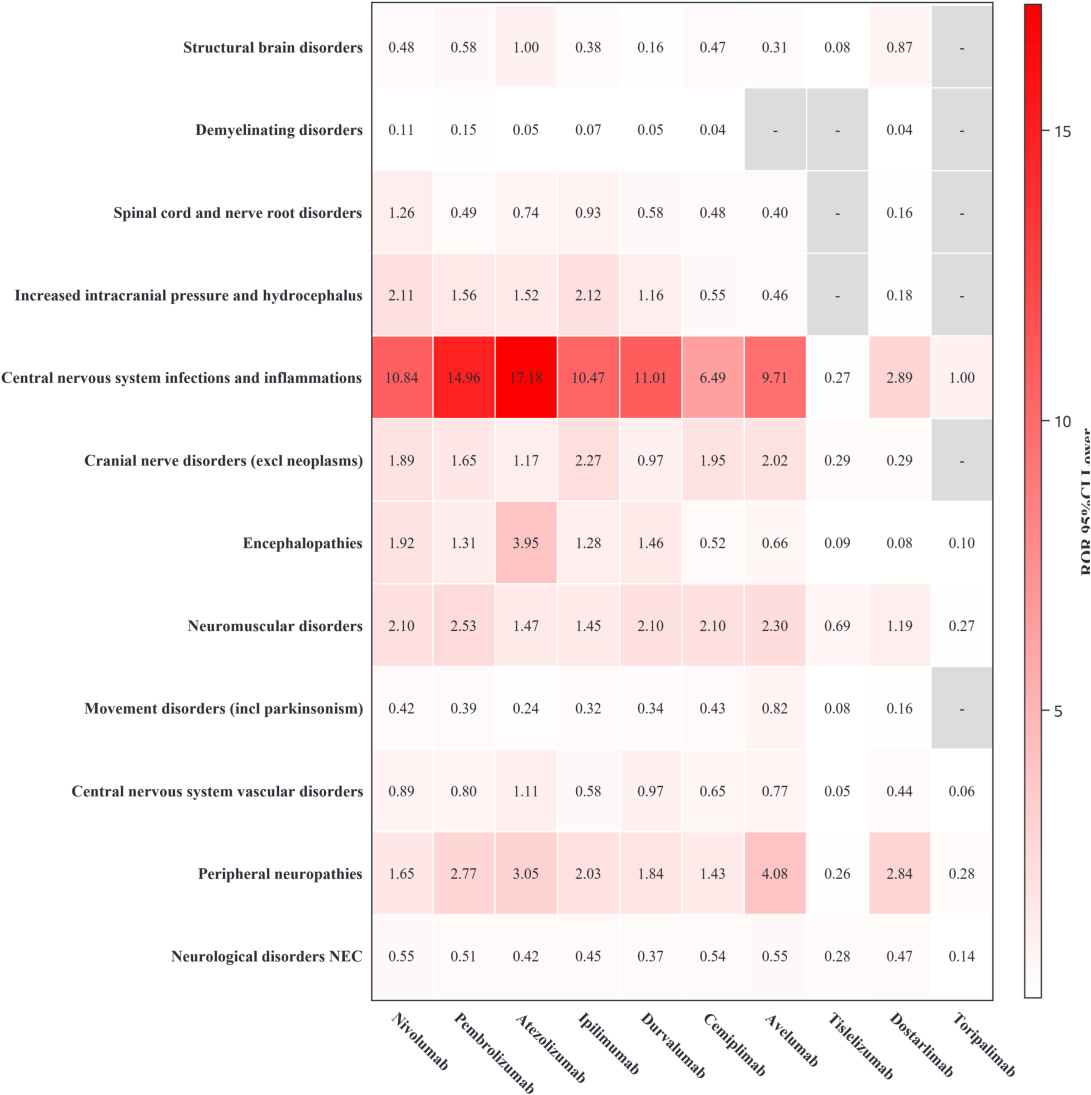
The signal intensity of 12 specific high-level group terms of neurological immune-related adverse event reports. This figure analyzes the Reporting Odds Ratio (ROR) signal intensity of 12 specific High-Level Group Terms (HLGTs) for neurotoxicity.

Eighteen HLGTs were involved in the neurological disorders SOC. Among the 50 PTs selected in this study, 12 were HLGTs with a cumulative total of 21,971 reports. Neurological disorders comprised six PTs (cumulative reports 9,168, 41.73%). Peripheral nervous system disorders comprised 12 PTs (cumulative reports 3,007, 13.69%). Central nervous system vascular disorders comprised four PTs, with cumulative reports of 2,822 (12.84%). Movement disorders (including Parkinson’s disease) comprised two PTs, with cumulative reports of 1,606 (7.31%). The remaining eight HLGTs comprised 26 PTs, with cumulative reports of 5,638 (24.43%) (**Figure 5**).Regarding signal strength, the signal strength for PD-1, PD-L1, and CTLA-4 drugs was mainly concentrated in the HLGT “Central nervous system infections and inflammations”: pembrolizumab, nivolumab, cemiplimab; atezolizumab, durvalumab, avelumab; ipilimumab. For these drugs, PTs with high signal strength in sex stratification, age stratification, and sensitivity analysis were consistent with central nervous system infections and inflammations. Dostarlimab showed a strong association with the HLGT “Peripheral nervous system disorders.” In age stratification, the PT with a higher signal in the elderly population was a peripheral nervous system disorder. In the sensitivity analysis excluding other drugs, the association with peripheral nervous system disorders remained high. Tislelizumab and toripalimab did not show significant signals, likely due to the low number of cases (**Figure 6**).

### 3.4 Combination therapy analysis

Different ICIs exhibit distinct neurotoxicity profiles. ICI monotherapy or combination therapy was associated with different reporting patterns of n-irAEs. The results of this study suggest that pembrolizumab combined with ipilimumab is associated with a signal for immune-mediated encephalitis. When combined with paclitaxel, peripheral neuropathy was the most frequently reported PT. Nivolumab had a strong association with immune-mediated encephalopathy; combination with ipilimumab was associated with a stronger signal for immune-mediated encephalopathy. Combination therapy with cabozantinib showed a signal for dysgeusia. Cemiplimab combined with paclitaxel showed a higher signal intensity for peripheral neuropathy. Tislelizumab monotherapy showed relatively clear signals for neurotoxic PTs; when combined with paclitaxel, it showed a higher signal for hypoesthesia. Dostarlimab monotherapy PT signals were not significant, but combined with paclitaxel and carboplatin, it showed stronger associations with peripheral neuropathy and syncope. Toripalimab combined with paclitaxel was associated with a signal for hypoesthesia. Atezolizumab had a strong association with autoimmune encephalitis; combination with avelumab was associated with a signal for Guillain-Barré syndrome; combination with etoposide showed a signal for paraneoplastic neurological syndromes; and combination with paclitaxel had a relatively high number of peripheral neuropathy cases. Durvalumab monotherapy was strongly associated with myasthenia gravis; combination with etoposide was associated with a signal for paraneoplastic neurological syndromes; and combination with cisplatin showed a higher signal for cerebral infarction. Avelumab monotherapy showed a high signal for myasthenia gravis; combination with levothyroxine showed a high signal for paraneoplastic encephalomyelitis. Ipilimumab combined with nivolumab was associated with a signal for immune-mediated optic neuritis and had a relatively high number of reports of altered state of consciousness (**Table 2**).

**Table 2:** Distribution of PT signals in immune checkpoint inhibitors combined with other drugs.

ICIs	Combined drugs	PT	Case N.	ROR_025_
Pembrolizumab	Paclitaxel	Neuropathy peripheral	209	9.84
	Ipilimumab	Immune-mediated encephalitis	3	141.15
Nivolumab	Cabozantinib	Taste disorder	216	56.80
	Ipilimumab	Immune-mediated encephalopathy	12	153.60
Cemiplimab	Carboplatin	Neuropathy peripheral	3	3.26
	Paclitaxel	Neuropathy peripheral	3	5.71
Tislelizumab	Paclitaxel	Hypoaesthesia	7	1.10
Dostarlimab	Carboplatin	Syncope	5	4.40
	Paclitaxel	Neuropathy peripheral	5	4.94
Toripalimab	Paclitaxel	Hypoaesthesia	3	13.14
Atezolizumab	Paclitaxel	Neuropathy peripheral	123	7.67
	Etoposide	Paraneoplastic neurological syndrome	6	343.38
	Avelumab	Guillain-Barre syndrome	6	37.41
Durvalumab	Cisplatin	Cerebral infarction	21	12.77
	Etoposide	Paraneoplastic neurological syndrome	4	409.53
Avelumab	Levothyroxine	Paraneoplastic encephalomyelitis	3	12705.43
	Axitinib	Cerebral infarction	3	3.01
Ipilimumab	Nivolumab	Altered state of consciousness	55	5.88
		Immune-mediated optic neuritis	3	201.86

ICI, immune checkpoint inhibitors; PT, preferred terms; ROR025, the lower limit of the 95% confidence interval of the Reporting Odds Ratio.

these findings are exploratory and should be interpreted with caution regarding the attribution to an additive immunological mechanism alone.

## 4 Discussion

Currently, monoclonal antibodies targeting PD-1, PD-L1, and CTLA-4 immune checkpoint inhibitors can enhance anti-tumor immune responses and improve survival rates in cancer patients (1, 8). While activating the immune system to attack tumor cells, ICIs may also disrupt the body’s immune tolerance to self-antigens, which can manifest as a series of immune-related adverse events (irAEs) (9). Among the various irAEs, neurological irAEs have a low incidence, and their mechanism is not fully understood (8); however, severe adverse events can lead to irreversible neurological damage or even death (10, 11). In this study, serious reports of ICI-related neurotoxicity accounted for 91.23% of cases, and life-threatening or fatal cases accounted for 26.61%. Therefore, neurotoxicity is a serious adverse event associated with ICIs that requires close monitoring. Pharmacovigilance studies play an important role in monitoring the safety of approved drugs. This study systematically analyzed the neurotoxic adverse events associated with 10 ICIs using the FAERS database, with the aim of providing evidence-based data to support clinical decision-making and regulatory risk management.

### 4.1 Relationship between demographic characteristics and neurotoxic adverse events

This study showed that the number of ADE reports was slightly higher in male patients than in female patients. However, some recent research results indicate minimal sex differences in immune-related adverse events (12, 13). In clinical practice, sex differences may not need consideration, but there may be sex tendencies for specific types of organ toxicity (14, 15). In this study, neurotoxicity primarily occurred in the elderly population, possibly related to the decline in immune system function in older patients, which might lead to reduced benefits from immunotherapy (16). In recent years, the indications for ICIs have expanded from lung cancer, melanoma, and renal cancer to other tumor types, such as gynecological and gastrointestinal cancers. The number of reported neurotoxic ADEs associated with ICIs has also continued to rise, indicating the gradual expansion of ICI use in clinical practice and the potential for neurotoxicity. Therefore, close monitoring of adverse events associated with ICIs in clinical practice is necessary for early diagnosis and treatment to avoid serious outcomes.

### 4.2 Significance of major neurological disease signal detection and mechanistic discussion

Through the analysis of signal association strength for 50 PTs across 10 ICIs, the central nervous system was primarily represented by immune-mediated encephalitis and encephalopathy in PTs, while the peripheral nervous system was primarily represented by immune-mediated myasthenia gravis and paraneoplastic neurological syndromes in PTs. These findings are largely consistent with those of previous studies (4, 17–19). ICIs achieve their therapeutic goals by relieving tumor-induced suppression of the immune system. In this process, T cell activation, especially CD8+ T cells, may recognize and attack neurons or glial cells, potentially contributing to neurotoxicity (20). Additionally, cytokine release, autoantibody-mediated neuronal damage, microglial overactivation, and changes in gut microbiota may also be important factors contributing to nervous system injury (21–25). PD-1 is primarily expressed on T cells, PD-L1 on tumor cells, and CTLA-4 is mainly expressed on activated T cells. Therefore, different types of ICIs show differences in the PT signal strength for neurotoxicity.

Among PD-1 inhibitors, the signal strength for immune-mediated myasthenia gravis was significant, especially for pembrolizumab, nivolumab, and tislelizumab, far exceeding that of other drugs. For Pembrolizumab and Nivolumab, this PT signal remained significant even after excluding combination drugs, possibly related to a significant increase in anti-acetylcholine receptor antibody positivity in patients treated with PD-1 inhibitors, suggesting that these drugs may be associated with the development or exacerbation of myasthenia gravis (26, 27). Cemiplimab showed signals for Lambert-Eaton myasthenic syndrome and myasthenia gravis, suggesting a potential association with neuromuscular diseases; however, the specific mechanism remains unclear, and current literature reports are scarce. Dostarlimab had a limited number of reports, with signals observed only for peripheral neuropathy. Further large-scale studies are required to collect more comprehensive data.

The PT signal profiles for PD-L1 inhibitors showed both overlaps and differences compared to those of PD-1 inhibitors. The PD-L1 drugs atezolizumab, durvalumab, and avelumab showed significant signals for paraneoplastic neurological syndromes, with relatively high ROR values observed for these PTs in this database. Atezolizumab also showed a signal for immune-mediated encephalitis, as did pembrolizumab, nivolumab, and ipilimumab. Furthermore, the signal strengths for atezolizumab, durvalumab, and avelumab varied among different patient groups. For example, in male patients, paraneoplastic neurological syndromes were the predominant signal for atezolizumab and durvalumab, whereas in female patients, immune-mediated encephalitis was more frequently observed. This pattern might be related to differences in immune responses and drug metabolism between the sexes (28). Additionally, in the age-stratified analysis, immune-mediated encephalitis was the predominant signal in young and middle-aged patients, whereas paraneoplastic neurological syndromes were more prominent in elderly patients. This pattern may reflect age-related differences in susceptibility to ICI-related toxicities (29). Therefore, patient age and sex should be considered in clinical practice.

The CTLA-4 inhibitor ipilimumab showed significant signals for immune-mediated encephalitis and myasthenia gravis. PD-1/PD-L1 inhibitors also showed signals for these PTs, with varying ROR values. Ipilimumab is often used in combination with other ICIs, potentially contributing to risk-superposition effects (30–32). Furthermore, dostarlimab showed weak signals for peripheral neuropathy-related PTs, and toripalimab did not show detectable PT signals, likely due to the limited number of reports. This absence of signals may be attributable to the limited sample size in the database, rather than indicating an absence of risk.

### 4.3 Relationship between combination therapy and neurotoxicity

In this study, ICIs were combined with immunotherapy, chemotherapy, and targeted drugs for antitumor treatment. Chemotherapy and targeted therapy can improve the tumor microenvironment, thereby enhancing immune responses and the efficacy of immunotherapy (33). This study found that pembrolizumab combined with ipilimumab showed a relatively significant signal for immune-mediated encephalitis, and nivolumab combined with ipilimumab showed a strong association with immune-mediated optic neuritis, consistent with previous research (18, 32). This might be related to excessive immune activation associated with the combination of CTLA-4 and PD-1/PD-L1 inhibitors, which may contribute to nervous system damage. Previous studies have shown that toxicity of dual immunotherapy may be higher than that of monotherapy (34). The neurotoxicity risk of ICIs combined with chemotherapy drugs warrants further attention. Atezolizumab and durvalumab combined with etoposide showed increased signal strength for paraneoplastic neurological syndromes (35, 36). This strong association may be related to the neurotoxicity of chemotherapy drugs. Etoposide can damage DNA in neurons, disrupting neural tissue integrity (37), whereas ICI-activated immune cells may further exacerbate inflammatory damage. Additionally, paclitaxel combined with pembrolizumab, cemiplimab, and toripalimab showed signals for peripheral neuropathy, although this was not highly significant. This may be related to the inherent peripheral neurotoxicity of paclitaxel and to PD-1/PD-L1 inhibitors inhibiting anti-nociceptive signaling, potentially exacerbating paclitaxel-associated neuropathic pain (38). Avelumab combined with levothyroxine showed a high signal for paraneoplastic encephalomyelitis. Such immune activation may be associated with the development of an autoimmune response against neural tissues. When the two agents are used in combination, they may exert a synergistic immunomodulatory effect, potentially contributing to autoimmune complications in the nervous system (39, 40). However, given that FAERS data are derived from spontaneous reports and thus susceptible to confounding factors, and that the sample sizes for some combination therapy findings are relatively small, the observed associations should be considered exploratory and require further verification through prospective studies or real-world data analyses.

## 5 Conclusion

In recent years, with the expanding indications and increased use of ICIs, the number of associated neurological diseases has gradually increased. This study comprehensively evaluated the association between ICIs and neurological diseases in real-world clinical practice. Future research should continue to elucidate the specific mechanisms underlying ICI-associated neurotoxic syndromes to reduce the risk of adverse events. The findings of this study are largely consistent with those of previous studies. Clinicians should be aware of the neurological toxicity characteristics of ICIs, enabling their early identification and diagnosis to improve the safety of ICI use in patients.

This study has the following limitations: (1) The FAERS database relies on spontaneous reporting, which may involve underreporting, low reporting, misreporting, missing data, or bias. (2) Some ICI drugs have been in the market for a short time, resulting in relatively scarce adverse event data. (3) Data primarily come from Europe, America, and Japan, with relatively little data from the Chinese population. (4) The FAERS data only included outcome information, without grading the severity of adverse events. (5) All findings are hypothesis-generating and reflect reporting disproportionality, not true risk. (6) ROR values cannot be used to directly compare the risk profiles of different ICIs.

In summary, this study mined data on ICI-associated neurotoxicity from the FAERS database and systematically analyzed the characteristics of these adverse events. It was found that among PD-1/PD-L1 drugs, pembrolizumab, nivolumab, and atezolizumab had high report counts and significant signals for immune-mediated myasthenia gravis, paraneoplastic neurological syndromes, and immune-mediated encephalitis/encephalopathy. CTLA-4 inhibitors also showed signals for immune-mediated encephalitis and myasthenia gravis. Therefore, close monitoring of changes in patient condition is essential when using drugs such as pembrolizumab and nivolumab in clinical practice. The first month after medication administration was the key monitoring period. By combining relevant guidelines and consensus recommendations, standardized detection, management, and treatment of neurotoxicity are recommended.

## Data Availability

Publicly available datasets were analyzed in this study. This data can be found here: https://fis.fda.gov/extensions/fpd-qde-faers/fpd-qde-faers.html.
